# Associations between meat consumption and all-cause and cause-specific mortality in middle-aged and older adults with frailty

**DOI:** 10.1016/j.jnha.2024.100191

**Published:** 2024-02-14

**Authors:** Jie Chen, Weihao Xu, Lintao Dan, Junhan Tang, Jirong Yue, Emiel O. Hoogendijk, Chenkai Wu

**Affiliations:** a Center for Global Health, Zhejiang University School of Medicine, Hangzhou, China; b Global Health Research Center, Duke Kunshan University, Kunshan, China; c Department of Cardiology, Guangdong Provincial Cardiovascular Institute, Guangdong Provincial People's Hospital, Guangdong Academy of Medical Sciences, Southern Medical University, Guangzhou, China; d Department of Geriatrics, Guangdong Provincial Geriatrics Institute, Guangdong Provincial People's Hospital, Guangdong Academy of Medical Sciences, Southern Medical University, Guangzhou, China; e Department of Geriatrics and National Clinical Research Center for Geriatrics, West China Hospital, Sichuan University, Chengdu, China; f Department of Epidemiology & Data Science and Department of General Practice, Location VU University Medical Center, Amsterdam UMC, Amsterdam, Netherlands; g Amsterdam Public Health Research Institute and Ageing & Later Life Research Program, Amsterdam UMC, Amsterdam, Netherlands; h Department of Psychiatry, University Medical Center Groningen, University of Groningen, Groningen, Netherlands

**Keywords:** Meat consumption, Diet, Frailty, Mortality

## Abstract

**Objectives:**

This study aimed to explore the associations between different types of meat consumption and mortality risk among people with frailty.

**Design:**

Longitudinal study.

**Setting and participants:**

We included 19,913 physically frail participants from the UK Biobank.

**Measurements:**

We used the validated brief food frequency questionnaire (FFQ) to measure meat consumption. Baseline diet data from 2006 to 2010 were collected, and participants were followed up until March 23, 2021. Cox proportional hazards regression models were conducted to examine the associations of meat consumption with mortality risk.

**Results:**

We identified 3,622 all-cause deaths, 1,453 cancer deaths, and 1,663 cardiovascular deaths during a median follow-up time of 11.2 years. Higher consumption of unprocessed poultry (per 25 g/day increment) was associated with a lower risk of all-cause mortality (hazard ratio [HR] 0.81, 95% confidence interval [CI] 0.75−0.88), cancer mortality (HR 0.84, 95% CI 0.74−0.96), and cardiovascular mortality (HR 0.72, 95% CI 0.63−0.81). Consumption of unprocessed red meat had a U-shaped relationship with mortality. Moderate consumption of unprocessed red meat 1.0−1.9 times/week was associated with a 14% (95% CI: 3 %–24%) lower risk of all-cause mortality than the lowest consumption frequency group (0−0.9 times/week). The hazard of cancer and CV mortality was also lower in the 1.0−1.9 times/week group, though the associations were not statistically significant. More frequent consumption of processed meat was associated with an increased risk of all-cause mortality (HR 1.20, 95% CI 1.07–1.34) and cardiovascular mortality (HR 1.20, 95% CI 1.02–1.42). Fish consumption was not associated with all types of mortality.

**Conclusions:**

Higher consumption of processed meat, not fish, was associated with increased all-cause and cardiovascular mortality. In contrast, higher consumption of unprocessed poultry and moderate consumption of unprocessed red meat was associated with reduced all-cause, cancer, and cardiovascular mortality. These findings warrant further investigation to establish optimal dietary patterns for frail individuals.

## 1 Introduction

Frailty is a complex, aging-related clinical syndrome characterized by decreased reserve to stressors, affecting approximately one in ten community-dwelling older adults worldwide [1]. Frail individuals have a compromised ability to maintain system homeostasis due to multi-system physiological dysregulation, leading to increased vulnerability to adverse outcomes and a substantial burden on healthcare resources [2]. Over the past two decades, a plethora of studies have attempted to develop measurement instruments for frailty, depict an epidemiological profile of frailty, and explore the causes and natural history of frailty [2]. Researchers have recently paid increasing attention to promoting the health and well-being of individuals with frailty. Becoming frail does not mean the end of life. Proper management of frailty could improve health and quality of life.

Nutrition plays a vital role in the development and progression of frailty. Two recently published clinical practice guidelines (CPGs) recommended protein supplementation for older adults with frailty to improve physical performance and strength [3,4]. Meat, including red meat, fish, and poultry, is a valuable energy source and contains essential nutrients, including high-value proteins and micronutrients. These nutrients are fundamental for maintaining skeletal muscle, improving physical function and strength, and preventing malnutrition among older adults [5]. Protein from different sources might have different effects on health. Higher consumption of unprocessed and processed red meat was associated with a higher risk of frailty, while protein intake from other sources showed health benefits [6,7]. The high content of saturated fatty acids has been linked to an increased risk of cardiovascular disease (CVD) [8]. Preservatives (e.g., nitrites) in processed meat might induce inflammation and oxidative stress, which are involved in the pathophysiological mechanisms of cardiometabolic disease [9,10]. The World Health Organization and many national dietary guidelines recommended limiting red and processed meat consumption to prevent cardiovascular and metabolic disorders among the general population [11,12].

Frail individuals have a higher need for protein because insufficient protein intake could lead to deterioration of frailty status and poor prognosis. However, little is known about how red meat and other primary dietary protein sources contribute to health among individuals with frailty. Moreover, it is unclear which types and frequencies of meat intake are associated with a notably higher mortality risk among the frail. High certainty evidence concerning nutrition intervention for managing frailty is limited. To fill out this gap, the present study aimed to examine the associations of consumption of different types of meat with all-cause mortality, cancer mortality, and cardiovascular mortality among frail individuals. The findings of this work would contribute to the development of food-based dietary guidelines for improving the health of people with frailty.

## 2 Methods

### 2.1 Data source

We leveraged data from the UK Biobank, an ongoing national prospective cohort study consisting of over half a million volunteers aged 40–69 years from 22 assessment centers in the UK between 2006 and 2010. Each participant was invited to attend the closest assessment center to complete a touch-screen questionnaire, a brief computer-assisted interview, physical measurements, and laboratory tests at baseline. The UK Biobank also enables the follow-up of medical and health-related records of individuals throughout the UK. Inpatient data was extracted from Hospital Episode Statistics for England, the Scottish Morbidity Record, and the Patient Episode Database for Wales. Cancer data were extracted through national cancer registries. Details of the study design and data collection have been documented elsewhere [13]. The present study is part of UK Biobank project 51450.

### 2.2 Analytic sample

**Fig. 1:**
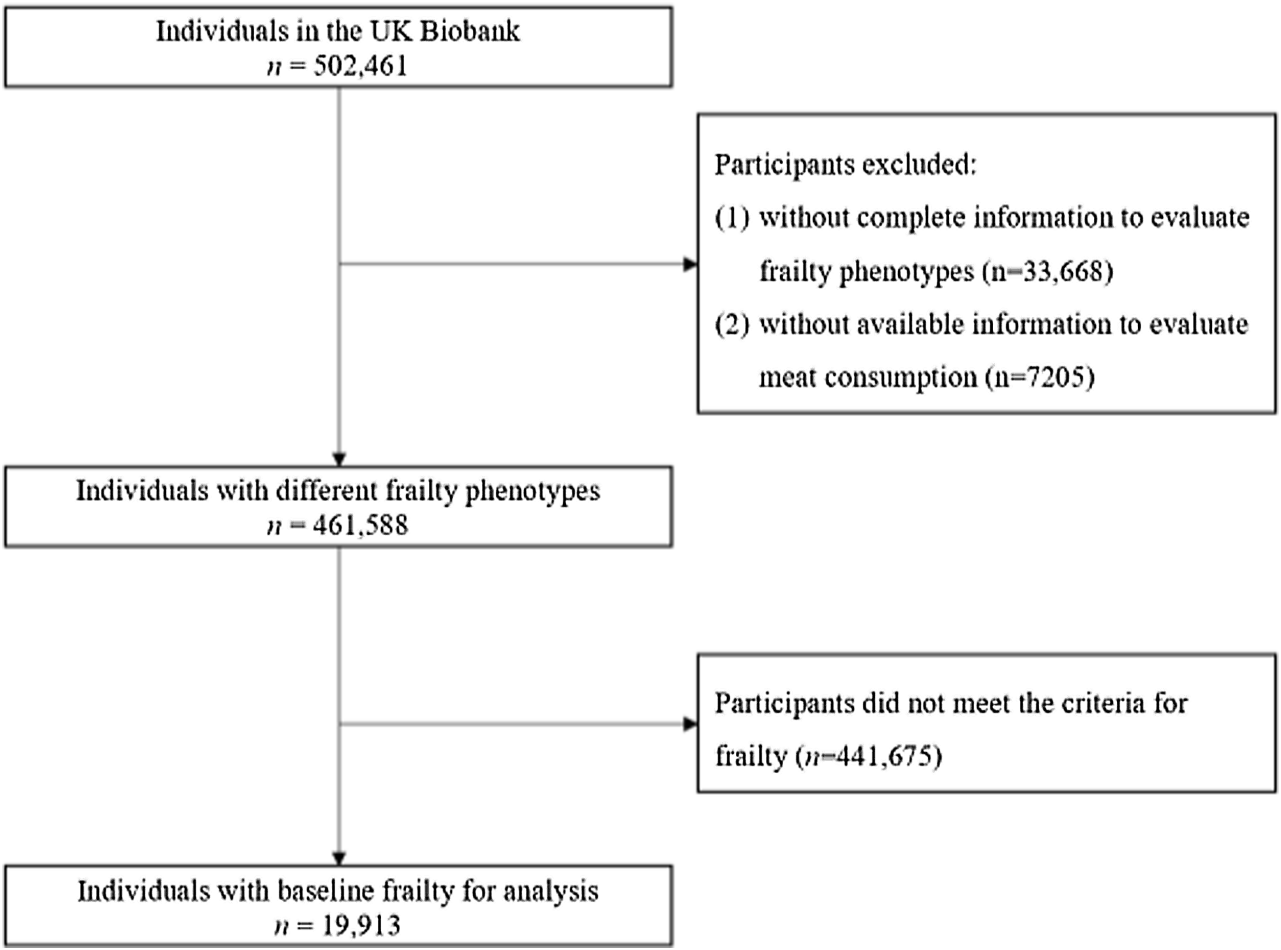
Flowchart of inclusion of study participants.

We excluded participants who (1) did not have sufficient information to measure physical frailty (n = 33,668) and (2) had no information to assess meat consumption (n = 7,205). The present study examined the association between meat consumption and mortality among the frail. We, therefore, further excluded individuals who were not frail at baseline (*n* = 441,675). The final analytic sample included 19,913 frail participants (Fig. 1). This study followed the Strengthening the Reporting of Observational Studies in Epidemiology (STROBE) guideline.

### 2.3 Frailty

Frailty was assessed using the physical frailty phenotype (PFP) approach developed by Fried and colleagues [14]. We used the modified version adapted to the available questions and measurements in the UK Biobank [15]. Details of the operational definitions of the five frailty criteria are provided in Table S1.

### 2.4 Mortality

The primary outcome was all-cause mortality. Death information was obtained through death certificates available in the National Health Service (NHS) Information Center for participants from England and Wales and the NHS Central Register, National Records of Scotland for participants from Scotland [16]. Details of the linkage procedure can be found at http://content.digital.nhs.uk/services [17]. Specific causes of death (i.e., cancer and cardiovascular) were defined using the following codes from the International Statistical Classification of Diseases and Related Health Problems, 10th Revision (ICD-10): cancer (C00-D48) and cardiovascular (I00-I79). Death data were available until March 23, 2021; Follow-up for death events was censored on this date or the date of death if this occurred earlier.

### 2.5 Meat consumption

We used the validated brief food frequency questionnaire (FFQ), part of the UK Biobank touch-screen questionnaire, to measure meat consumption. The FFQ included 47 questions about diet, asking about the average consumption frequency of principal foods, food groups, and drinking habits over the past year to obtain pertinent dietary information at the recruitment assessment center visit. Details of the operational assessment of meat consumption were provided in eMethod and Table S2.

### 2.6 Other variables

Socio-demographic characteristics included age at recruitment (years), sex, ethnicity (White and Others), education (below college and college or above), and the Townsend deprivation index (TDI)—a commonly used measure of material deprivation in UK Biobank. Lifestyle variables included smoking status, alcohol use, physical activity, body mass index (BMI), and other major food groups (grain products, vegetables, and fruit). Smoking status was classified into current or previous smoking and never smoking. Participants were asked about the consumption of wine, beer, spirit, and other alcoholic drinks. We used the self-reported frequency and volume of alcohol consumption to measure alcohol use. Participants were classified into none to moderate (<14 grams per day for women and <28 grams per day for men) and high consumption [18]. BMI was calculated as body weight in kilograms divided by standing height in meters squared.

### 2.7 Statistical analysis

Baseline socio-demographic and lifestyle characteristics were summarized for all frail participants. Means (SDs) and numbers (percentages) were used to describe continuous and categorical variables, respectively. We estimated hazard ratios (HR) and 95% confidence intervals (CI) between meat consumption and risk for all-cause and cause-specific mortality using Cox proportional hazards regression models with 0−0.9 times/week of meat intakes as the reference categories. We also analyzed the effects of a fixed intake increase (25 g/day) on the outcomes. We calculated participants’ survival time in person-years from their age at baseline recruitment to death, loss-to-follow-up, or administrative censoring, whichever came first. The fully adjusted model included age (continuous), sex (male or female), ethnicity (white or others), Townsend deprivation index (continuous), education level (college and above or below college), smoking (previous/current smokers or never smoked), BMI (continuous), alcohol consumption (none to moderate or high), intake of grain products, vegetables, fruit (continuous) and sugary sweetened beverage consumption (yes/no).

We conducted several subgroup analyses stratified by age (<60 or ≥60 years), sex (male or female), and BMI (≤25 kg/m^2^ or >25 kg/m^2^) using the fully adjusted model to test potential effect modifications. We conducted several sensitivity analyses. First, we excluded participants who died within the first year and the first two years of follow-up. Second, we imputed missing values using multiple imputations. Third, we additionally adjusted for the Charlson Comorbidity Index (CCI). With substitution analysis, we estimated the effect of replacing one time/week intake of processed meat or unprocessed red meat consumption for an equal exchange of 1 time/week intake of other sources of meat (including fish, unprocessed poultry, unprocessed red meat, or processed meat) and cheese on all-cause mortality risk. We simultaneously included all types of meat but omitted the type of meat of interest together with the covariates listed above for the substitution analysis. All analyses were conducted using R 4.1.1. Two-sided P values <0.05 were considered statistically significant.

## 3 Results

### 3.1 Baseline characteristics

The baseline characteristics of the included participants are shown in Table 1. Of the 19,913 frail participants, the mean age was 58.12 years (SD = 7.64 years), and 11,823 (59.4%) were female. During 222,463 person-years of follow-up (mean follow-up 11.2 years), 3,622 all-cause deaths, 1,453 cancer deaths, and 1,663 cardiovascular deaths were documented. The proportions of participants who consumed fish, unprocessed poultry, unprocessed red meat, or processed meat 0–0.9 times/week were 13.0%, 19.4%, 37.5%, and 11.8%, respectively.

**Table 1:** Baseline characteristics of frail adults in the UK Biobank.

Characteristics	N = 19,913
Age at recruitment, year, mean (SD)	58.12 (7.64)
Sex, n (%)	
Female	11,823 (59.4)
Male	8,090 (40.6)
Townsend deprivation index, mean (SD)	0.38 (3.55)
Education, n (%)	
Below college	16,257 (83.0)
College and above	3,325 (17.0)
Ethnicity, n (%)	
White	17,677 (89.2)
Others	2,141 (10.8)
Waist circumstances, cm, mean (SD)	97.06 (16.09)
Body mass index, kg/m^2^, mean (SD)	30.20 (6.65)
Smoking status, n (%)	
Previous/current smokers	10,950 (55.3)
Never smoked	8,846 (44.7)
Alcohol consumption, n (%)	
None to moderate	2,809 (14.1)
High	17,067 (85.9)
Charlson Comorbidity Index, mean (SD)	0.79 (1.51)
Fish, n (%)	
0−0.9 time/week	2,582 (13.0)
1.0−1.9 times/week	8,229 (41.3)
2.0−4.0 times/week	7,670 (38.5)
>4.0 times/week	1,432 (7.2)
Unprocessed poultry, n (%)	
0−0.9 time/week	3,856 (19.4)
1.0−1.9 times/week	6,818 (34.2)
2.0−4.0 times/week	8,525 (42.8)
>4.0 times/week	714 (3.6)
Unprocessed red meat, n (%)	
0−0.9 time/week	7,477 (37.5)
1.0−1.9 times/week	5,377 (27.0)
2.0−4.0 times/week	5,860 (29.4)
>4.0 times/week	1,199 (6.0)
Processed meat, n (%)	
0−0.9 time/week	2,341 (11.8)
1.0−1.9 times/week	7,512 (37.7)
2.0−4.0 times/week	7,971 (40.0)
>4.0 times/week	2,089 (10.5)

### 3.2 Meat consumption and mortality

The associations between meat consumption and mortality among frail participants are shown in Table 2. Frail individuals who consumed unprocessed poultry more frequently had a lower hazard of all-cause mortality (HRs = 0.89, 0.83, 0.66 for 1.0−1.9 times/week, 2.0−4.0 times/week and >4.0 times/week compared to the 0−0.9 time/week group, respectively; *P*-trend for each 25 g increment<0.001). Higher consumption of unprocessed poultry was associated with a lower hazard of cancer mortality (HRs = 0.91, 0.82, 0.81 for 1.0−1.9 times/week, 2.0−4.0 times/week and >4.0 times/week compared to the 0−0.9 time/week group, respectively; *P*-trend for each 25 g increment = 0.013) and cardiovascular mortality (HRs = 0.84, 0.73, 0.52 for 1.0−1.9 times/week, 2.0−4.0 times/week and >4.0 times/week compared to the 0−0.9 time/week group, respectively; *P*-trend for each 25 g increment<0.001). On the contrary, higher consumption of processed meat was associated with a higher hazard of all-cause mortality (HRs = 1.04, 1.11, 1.21 for 1.0−1.9 times/week, 2.0−4.0 times/week and >4.0 times/week compared to the 0−0.9 time/week group, respectively; *P*-trend for each 25 g increment = 0.002) and cardiovascular mortality (HRs = 1.15, 1.16, 1.25 for 1.0−1.9 times/week, 2.0−4.0 times/week and >4.0 times/week compared to the 0−0.9 time/week group, respectively; *P*-trend for each 25 g increment = 0.027). We found a U-shape relationship between unprocessed red meat consumption and mortality. Consumption of unprocessed red meat 1.0−1.9 times/week was associated with a 14% (95% CI: 3%, 24%) lower hazard of all-cause mortality than the lowest consumption frequency group (0−0.9 times/week). The hazard of cancer and cardiovascular mortality was also lower in the 1.0−1.9 times/week group, though the associations were not significant. When modeled continuously, each 25 g increment in consumption of unprocessed red meat was associated with a 7% (95% CI: 1%, 14%) and 16% (95% CI: 5%, 28%) higher hazard of all-cause and cardiovascular mortality among the frail, respectively. We did not observe a significant association between fish (HR = 0.99, 95% CI: 0.85–1.16; *P*-trend for each 25 g increment = 0.791) and total meat (HR = 0.91, 95% CI: 0.74–1.11; *P*-trend for each 25 g increment = 0.353) consumption with any type of mortality.

**Table 2:** Association of meat intake with all-cause, cancer, and cardiovascular mortality among frail adults in the UK Biobank.

		All-cause mortality			Cancer mortality			Cardiovascular mortality		
	Mean intake (g/day)^2^	Cases/PYs	HR (95% CI)	*P*	Cases/PYs	HR (95% CI)	*P*	Cases/PYs	HR (95% CI)	*P*
Fish										
0−0.9 time/week	7	426/29,071	Ref.		155/29,071	Ref.		197/29,071	Ref.	
1.0−1.9 times/week	28	1470/91,896	0.97 (0.87, 1.08)	0.577	587/91,896	1.04 (0.87, 1.25)	0.647	675/91,896	0.95 (0.81, 1.12)	0.560
2.0−4.0 times/week	45	1457/85,661	0.97 (0.87, 1.09)	0.640	613/85,661	1.12 (0.93, 1.35)	0.224	668/85,661	0.94 (0.79, 1.11)	0.443
>4.0 times/week	72	269/15,975	0.99 (0.85, 1.16)	0.916	98/15,975	1.01 (0.78, 1.31)	0.938	123/15,975	0.93 (0.74, 1.17)	0.526
per 25 g/day			0.99 (0.92, 1.07)	0.791		1.03 (0.91, 1.16)	0.614		0.95 (0.85, 1.07)	0.414
Unprocessed poultry										
0−0.9 time/week	12	811/42,508	Ref.		319/42,508	Ref.		390/42,508	Ref.	
1.0−1.9 times/week	28	1345/75,757	0.89 (0.81, 0.98)	0.012	554/75,757	0.91 (0.79, 1.05)	0.215	630/75,757	0.84 (0.74, 0.96)	0.011
2.0−4.0 times/week	39	1384/96,154	0.83 (0.76, 0.91)	<0.001	542/96,154	0.82 (0.70, 0.94)	0.006	610/96,154	0.73 (0.64, 0.84)	<0.001
>4.0 times/week	63	82/8,184	0.66 (0.52, 0.83)	<0.001	38/8,184	0.81 (0.57, 1.14)	0.221	33/8,184	0.52 (0.36, 0.75)	<0.001
per 25 g/day			0.81 (0.75, 0.88)	<0.001		0.84 (0.74, 0.96)	0.013		0.72 (0.63, 0.81)	<0.001
Unprocessed red meat										
0−0.9 time/week	6	367/26,438	Ref.		141/26,438	Ref.		147/26,438	Ref.	
1.0−1.9 times/week	32	1182/84,708	0.86 (0.76, 0.97)	0.018	483/84,708	0.91 (0.75, 1.10)	0.335	533/84,708	0.95 (0.78, 1.15)	0.598
2.0−4.0 times/week	45	1595/88,575	1.01 (0.89, 1.14)	0.915	646/88,575	1.06 (0.87, 1.30)	0.534	748/88,575	1.13 (0.93, 1.36)	0.227
>4.0 times/week	60	478/22,882	1.10 (0.95, 1.27)	0.207	183/22,882	1.13 (0.90, 1.43)	0.300	235/22,882	1.25 (1.00, 1.56)	0.046
per 25 g/day			1.07 (1.01, 1.14)	0.033		1.09 (0.99, 1.21)	0.095		1.16 (1.05, 1.28)	0.003
Processed meat										
0−0.9 time/week	12	1128/84,696	Ref.		473/84,696	Ref.		470/84,696	Ref.	
1.0−1.9 times/week	20	971/60,168	1.04 (0.95, 1.14)	0.356	383/60,168	0.97 (0.85, 1.12)	0.717	464/60,168	1.15 (1.01, 1.32)	0.034
2.0−4.0 times/week	27	1245/64,677	1.11 (1.02, 1.22)	0.016	498/64,677	1.09 (0.95, 1.25)	0.204	594/64,677	1.16 (1.02, 1.32)	0.025
>4.0 times/week	37	278/13,062	1.21 (1.05, 1.39)	0.008	99/13,062	1.07 (0.85, 1.35)	0.540	135/13,062	1.25 (1.02, 1.53)	0.030
per 25 g/day			1.20 (1.07, 1.34)	0.002		1.15 (0.97, 1.38)	0.116		1.20 (1.02, 1.42)	0.027
Total meat										
0−2.9 times/week	3	398/30,476	Ref.		166/30,476	Ref.		176/30,476	Ref.	
3.0−4.9 times/week	36	876/53,049	0.84 (0.63, 1.14)	0.265	359/53,049	0.70 (0.44, 1.11)	0.130	382/53,049	0.99 (0.63, 1.55)	0.951
5.0−6.9 times/week	73	1040/66,715	0.87 (0.70, 1.06)	0.171	434/66,715	0.75 (0.55, 1.03)	0.076	499/66,715	0.93 (0.67, 1.30)	0.674
≥7.0 times/week	101	1308/72,363	0.91 (0.74, 1.11)	0.353	494/72,363	0.79 (0.59, 1.07)	0.131	606/72,363	0.99 (0.72, 1.37)	0.956
per 25 g/day			1.00 (0.96, 1.04)	0.949		0.99 (0.93, 1.06)	0.821		1.01 (0.95, 1.08)	0.678

Abbreviations: PY, person-year; HR, hazard ratio; CI, confidence interval.

### 3.3 Substitution analyses

In the substitution analyses, replacing processed meat with oily fish was associated with 5% reduced risk of all-cause mortality; replacing processed meat with unprocessed poultry was associated with a significantly lower hazard of all types of mortality; the percentage in reduction of hazard was 9% for all-cause mortality, 7% for cancer-specific mortality, and 13% for cardiovascular mortality (Table 3). For the replacement of unprocessed red meat, the percentage reduction in the hazard of all-cause mortality was 3% for fish, 6% for oily fish, 10% for unprocessed poultry, and 4% for cheese. Additionally, the percentage reduction in the hazard of cancer-specific mortality was 9% for unprocessed poultry and 7% for cheese, and the reduction in the hazard of cardiovascular mortality was 5% for fish, 13% for unprocessed poultry, and 5% for cheese.

**Table 3:** Hazard rations of all-cause, cancer, and cardiovascular mortality for the replacement of 1 time per week of different types of meat for unprocessed and processed red meat among frail adults in the UK Biobank.

	All-cause mortality		Cancer-specific mortality		Cardiovascular-specific mortality	
	HR (95% CI)	*P*	HR (95% CI)	*P*	HR (95% CI)	*P*
Replacement for processed meat						
Fish	0.97 (0.94, 1.00)	0.060	0.99 (0.94, 1.03)	0.561	0.96 (0.92, 1.00)	0.057
Oily fish	0.95 (0.91, 0.99)	0.012	0.98 (0.92, 1.04)	0.483	0.96 (0.90, 1.01)	0.139
Non-oily fish	1.00 (0.96, 1.04)	0.958	1.00 (0.93, 1.07)	0.902	0.96 (0.90, 1.03)	0.242
Unprocessed poultry	0.91 (0.88, 0.94)	<0.001	0.93 (0.88, 0.98)	0.008	0.87 (0.83, 0.92)	<0.001
Unprocessed red meat	1.01 (0.98, 1.04)	0.692	1.02 (0.97, 1.07)	0.489	1.01 (0.96, 1.06)	0.702
Beef	1.01 (0.96, 1.07)	0.747	1.05 (0.97, 1.15)	0.246	1.01 (0.93, 1.09)	0.794
Pork	1.00 (0.95, 1.06)	0.984	1.03 (0.94, 1.12)	0.551	0.98 (0.91, 1.06)	0.633
Mutton	1.01 (0.97, 1.05)	0.656	0.99 (0.93, 1.07)	0.856	1.03 (0.97, 1.09)	0.382
Other protein source						
Cheese	0.97 (0.94, 1.01)	0.103	0.96 (0.91, 1.01)	0.088	0.97 (0.92, 1.01)	0.155
Replacement for unprocessed red meat						
Fish	0.97 (0.94, 0.99)	0.016	0.97 (0.93, 1.01)	0.161	0.95 (0.91, 0.99)	0.015
Oily fish	0.94 (0.91, 0.98)	0.003	0.96 (0.90, 1.02)	0.193	0.95 (0.90, 1.00)	0.062
Non-oily fish	0.99 (0.95, 1.03)	0.698	0.98 (0.92, 1.04)	0.509	0.95 (0.90, 1.01)	0.127
Unprocessed poultry	0.90 (0.87, 0.93)	<0.001	0.91 (0.86, 0.96)	0.001	0.87 (0.82, 0.91)	<0.001
Processed meat	0.99 (0.96, 1.03)	0.692	0.98 (0.93, 1.03)	0.489	0.99 (0.95, 1.04)	0.702
Other protein source						
Cheese	0.96 (0.94, 0.99)	0.008	0.93 (0.89, 0.97)	0.002	0.95 (0.91, 0.99)	0.022

Abbreviations: HR, hazard ratio; CI, confidence interval.

### 3.4 Subgroup analyses

We observed similar associations of different types of meat with all-cause mortality in analyses stratified by age (P-values for interactions >0.05 for all; Table 4). There was a significant interaction between unprocessed red meat consumption frequency and sex for all-cause mortality (P for interaction = 0.022). Consumption of unprocessed red meat for each 25 g increment was associated with a higher hazard of all-cause mortality among males, while the association was almost null among females. In addition, the association between more frequent consumption of processed meat and a higher hazard of all-cause mortality was stronger among individuals with a BMI ≤ 25 kg/m^2^ than those with a BMI > 25 kg/m^2^. A similar interaction was found between total meat consumption frequency and BMI for all-cause mortality.

**Table 4:** Association of meat consumption with all-cause mortality among frail adults by age, sex, and body mass index.

	Age <60 years		Age ≥60 years		Male		Female		BMI ≤ 25 kg/m^2^		BMI > 25 kg/m^2^	
	HR (95% CI)	*P*	HR (95% CI)	*P*	HR (95% CI)	*P*	HR (95% CI)	*P*	HR (95% CI)	*P*	HR (95% CI)	*P*
Fish												
0−0.9 time/week	Ref		Ref	0.518^a^	Ref		Ref	0.503 a	Ref		Ref	0.622 a
1.0−1.9 times/week	1.04 (0.87, 1.25)	0.641	0.99 (0.86, 1.14)	0.913	1.06 (0.91, 1.23)	0.465	0.86 (0.73, 1.02)	0.083	0.97 (0.78, 1.20)	0.754	0.97 (0.85, 1.10)	0.622
2.0−4.0 times/week	1.08 (0.90, 1.31)	0.411	1.02 (0.88, 1.17)	0.819	1.00 (0.86, 1.16)	0.974	0.94 (0.79, 1.11)	0.468	0.94 (0.75, 1.18)	0.595	0.98 (0.86, 1.12)	0.749
>4.0 times/week	1.18 (0.90, 1.56)	0.227	0.99 (0.82, 1.21)	0.956	0.96 (0.77, 1.18)	0.685	1.03 (0.82, 1.31)	0.781	0.97 (0.71, 1.32)	0.834	0.98 (0.82, 1.18)	0.822
per 25 g/day	1.08 (0.95, 1.24)	0.226	1.00 (0.91, 1.10)	0.999	0.96 (0.86, 1.06)	0.389	1.03 (0.92, 1.16)	0.598	0.98 (0.84, 1.14)	0.799	0.99 (0.90, 1.08)	0.743
Unprocessed poultry												
0−0.9 time/week	Ref		Ref	0.075^a^	Ref		Ref	0.676^a^	Ref		Ref	0.244^a^
1.0−1.9 times/week	0.79 (0.66, 0.93)	0.006	0.91 (0.82, 1.01)	0.090	0.90 (0.80, 1.01)	0.070	0.87 (0.75, 1.01)	0.067	1.03 (0.86, 1.23)	0.782	0.87 (0.78, 0.96)	0.008
2.0−4.0 times/week	0.71 (0.60, 0.84)	<0.001	0.83 (0.74, 0.92)	0.001	0.81 (0.72, 0.91)	0.001	0.85 (0.73, 0.98)	0.022	0.95 (0.79, 1.14)	0.598	0.80 (0.72, 0.89)	<0.001
>4.0 times/week	0.47 (0.32, 0.71)	<0.001	0.69 (0.52, 0.91)	0.010	0.62 (0.45, 0.86)	0.004	0.70 (0.50, 0.97)	0.032	0.81 (0.50, 1.33)	0.410	0.62 (0.48, 0.81)	<0.001
per 25 g/day	0.69 (0.59, 0.80)	<0.001	0.82 (0.74, 0.91)	<0.001	0.79 (0.71, 0.89)	<0.001	0.83 (0.73, 0.94)	0.005	0.93 (0.79, 1.10)	0.411	0.79 (0.71, 0.87)	<0.001
Unprocessed red meat												
0−0.9 time/week	Ref		Ref	0.617^a^	Ref		Ref	**0.022**^a^	Ref		Ref	0.443^a^
1.0−1.9 times/week	0.87 (0.71, 1.07)	0.197	0.89 (0.77, 1.04)	0.156	0.83 (0.70, 1.00)	0.045	0.90 (0.76, 1.07)	0.240	0.85 (0.67, 1.07)	0.157	0.87 (0.75, 1.00)	0.052
2.0−4.0 times/week	1.02 (0.82, 1.25)	0.887	1.07 (0.91, 1.24)	0.417	1.04 (0.87, 1.24)	0.647	0.97 (0.82, 1.16)	0.767	1.05 (0.83, 1.32)	0.687	0.99 (0.85, 1.14)	0.872
>4.0 times/week	1.16 (0.89, 1.50)	0.263	1.17 (0.98, 1.40)	0.090	1.17 (0.96, 1.43)	0.118	1.01 (0.81, 1.26)	0.945	1.00 (0.75, 1.33)	0.973	1.13 (0.95, 1.34)	0.158
per 25 g/day	1.07 (0.95, 1.19)	0.270	1.12 (1.04, 1.22)	0.004	1.16 (1.05, 1.27)	0.002	0.99 (0.91, 1.09)	0.873	1.07 (0.95, 1.21)	0.252	1.07 (1.00, 1.16)	0.063
Processed meat												
0−0.9 time/week	Ref		Ref	0.578^a^	Ref		Ref	0.993^a^	Ref		Ref	**0.016**^a^
1.0−1.9 times/week	1.02 (0.87, 1.21)	0.785	1.06 (0.95, 1.17)	0.316	1.02 (0.89, 1.16)	0.796	1.07 (0.94, 1.21)	0.294	1.19 (0.99, 1.43)	0.058	1.00 (0.90, 1.11)	0.968
2.0−4.0 times/week	1.14 (0.97, 1.34)	0.108	1.12 (1.01, 1.24)	0.035	1.10 (0.97, 1.24)	0.131	1.13 (0.99, 1.29)	0.071	1.24 (1.03, 1.48)	0.020	1.07 (0.97, 1.19)	0.166
>4.0 times/week	1.07 (0.83, 1.37)	0.623	1.26 (1.06, 1.49)	0.007	1.17 (0.99, 1.38)	0.068	1.26 (0.95, 1.67)	0.106	1.24 (0.94, 1.63)	0.126	1.17 (1.00, 1.38)	0.055
per 25 g/day	1.17 (0.95, 1.44)	0.137	1.22 (1.06, 1.39)	0.004	1.18 (1.02, 1.36)	0.024	1.22 (1.02, 1.47)	0.031	1.24 (0.99, 1.56)	0.066	1.17 (1.03, 1.33)	0.019
Total meat												
0−2.9 times/week	Ref		Ref	0.188^a^	Ref		Ref	0.479^a^	Ref		Ref	0.024^a^
3.0−4.9 times/week	0.79 (0.50, 1.27)	0.334	0.90 (0.61, 1.33)	0.587	0.95 (0.60, 1.49)	0.820	0.78 (0.52, 1.17)	0.224	0.76 (0.45, 1.30)	0.320	0.87 (0.61, 1.25)	0.452
5.0−6.9 times/week	0.73 (0.53, 1.00)	0.050	1.02 (0.78, 1.35)	0.866	0.87 (0.62, 1.22)	0.425	0.88 (0.68, 1.15)	0.348	0.95 (0.66, 1.35)	0.758	0.84 (0.65, 1.08)	0.166
≥7.0 times/week	0.73 (0.54, 0.99)	0.043	1.07 (0.82, 1.41)	0.607	0.94 (0.68, 1.31)	0.728	0.90 (0.70, 1.16)	0.421	1.07 (0.76, 1.50)	0.711	0.86 (0.67, 1.10)	0.228
per 25 g/day	0.95 (0.89, 1.01)	0.085	1.03 (0.98, 1.09)	0.176	1.02 (0.96, 1.08)	0.518	0.98 (0.93, 1.04)	0.559	1.06 (0.98, 1.13)	0.139	0.98 (0.94, 1.03)	0.512

Abbreviations: BMI, body mass index; HR, hazard ratio; CI, confidence interval.

a P for interaction.

### 3.5 Sensitivity analyses

The observed results remained essentially unchanged in several sensitivity analyses. The associations between unprocessed poultry, processed meat, and unprocessed red meat consumption and all-cause mortality persisted when we excluded death cases in the first 1-year and 2-year follow-up periods (Table S3). We also observed consistent associations of unprocessed poultry, processed meat, and unprocessed red meat consumption with all-cause mortality when missing values were reprocessed using multiple imputations, or CCI was additionally adjusted (Table S3).

## 4 Discussion

Based on a large cohort study of nearly 20,000 individuals with frailty, we showed that more frequent intake of unprocessed poultry was associated with a reduced risk of all-cause, cancer, and cardiovascular mortality, whereas more frequent intake of processed meat was associated with increased all-cause and cardiovascular mortality. In addition, we found a U-shape relationship between unprocessed red meat consumption frequency and mortality, with a moderate consumption frequency (1.0−1.9 times/week) being associated with the lowest mortality. Moreover, substituting processed meat or unprocessed red meat with fish was associated with reduced all-cause mortality; replacement of processed meat or unprocessed red meat with unprocessed poultry was associated with lower all-cause, cancer, and cardiovascular mortality. Taken together, these findings had significant implications for CPGs and dietary guidelines for individuals living with frailty.

The harmful health effects of processed meat intake have been well documented in previous studies that examined the association between different types of meat intake and health outcomes among the general population [19,20]. A recent meta-analysis by Zeraatkar et al. reported that a reduction of 3 servings per week of processed red meat was associated with an 8% and 10% lower risk of all-cause and cardiovascular mortality, respectively [21]. Our study, which focused exclusively on middle-aged and older adults living with frailty, found similar associations between processed meat and mortality outcomes. Although frail individuals have an increased need for protein, the harmful effects of the detrimental components in processed meat might outweigh the benefits of protein supplementation.

Our finding of the association between unprocessed red meat intake and mortality risk was not in line with existing evidence revealed among the general population. Zeraatkar et al. reported that a reduction in unprocessed red meat of 3 servings per week was associated with 7% and 10% lower risk of all-cause and cardiovascular mortality, respectively [21]. We found that a moderate consumption frequency of unprocessed red meat (1.0−1.9 times/week) was associated with the lowest mortality among frail individuals. However, when modeled continuously, a higher consumption was related to a higher mortality risk. Unprocessed red meat has many high-quality proteins essential for maintaining physical health; however, it contains high amounts of saturated fatty acids, which might increase the risk of CVD [22]. A subtle balance point might exist between the harmful and beneficial effects of unprocessed red meat consumption among individuals with frailty. The beneficial effect of unprocessed red meat might be more salient among the frail than the general population.

Previous meta-analyses found that higher poultry intake was associated with reduced risk of all-cause and cancer mortality but not cardiovascular mortality in the general population [23,24]. Our study showed similar associations of poultry consumption with mortality, in which a higher intake of unprocessed poultry was associated with a lower risk of all-cause and cancer mortality among people with frailty. In addition, we found that a higher intake of unprocessed poultry was associated with reduced cardiovascular mortality. This finding did not contradict the previous meta-analysis because we focused on unprocessed poultry. In contrast, previous studies mainly investigated the health effects of total poultry intake, including processed and unprocessed poultry. Interestingly, a large prospective cohort study including 536,969 general people found that a higher intake of processed white meat (poultry cold cuts, low-fat sausages, and low-fat hotdogs made from poultry) was associated with decreased mortality risk [25]. Future studies are still warranted for a more comprehensive understanding of the health effects of processed and unprocessed poultry consumption. We did not observe an association between fish intake and mortality among individuals living with frailty. However, the beneficial health effects of fish consumption in the general population have been documented [26,27].

In our substitution analyses, our finding that replacing processed meat or unprocessed red meat with unprocessed poultry or fish was associated with a reduced mortality risk was consistent with previous studies [23,28]. A previous meta-analysis indicated substituting red or processed meat with poultry was inversely associated with all-cause mortality and risk of CVD [23]. Another meta-analysis found that replacing total red meat with fish/seafood was associated with a lower risk of all-cause mortality [28]. Our results contributed to the literature by showing that poultry and fish could be a healthier alternative to red and processed meat among frail individuals.

We observed similar associations in analysis stratified by age. A previous study also showed no age difference in the associations between meat consumption and all-cause mortality among the general population [29]. We also found several subgroup differences, including the findings that unprocessed red meat intake was significantly positively associated with increased mortality risk only in males and the BMI differences in the association between processed meat consumption and mortality risk. The reasons for the differences were unclear, and these subgroup findings warrant future investigations.

Nutritional guidelines are available for the general population; however, there are only a few CPGs that provide detailed recommendations about nutrition interventions, especially protein intake for frail individuals. The task force of the International Conference of Frailty and Sarcopenia Research developed CPGs that suggested protein supplementation can be considered for frail individuals with weight loss or undernutrition [4]. Similarly, the Canadian Frailty Network recently developed the CPGs, recommending that protein-fortified foods/supplements be considered for older adults with frailty or pre-frailty [3]. However, the current CPGs for frail individuals mentioned no detailed protein intake recommendations. Even so, these guidelines are mainly based on expert opinion. The level of evidence is low or very low. We need much more research on optimal dietary components for people with frailty because current clinical guidelines are mainly based on expert consensus because of the lack of an evidence base. To our knowledge, this is the first study examining the associations of different types of meat consumption on the mortality risk among frail individuals. There are several strengths in our study. First, we took advantage of the large sample size and well-administrated cohort from the UK Biobank, which enabled a series of analyses on the specific population with frailty. Second, an adequate number of death cases defined based on the NHS death records allowed us to assess the associations with acceptable statistical power. Third, potential confounding effects were considered seriously and dealt with through multiple adjustments and sensitivity analyses. Our study also has several limitations. First, most of the participants included were of European ancestry, so our findings may not be generalizable to other populations. In addition, measurement error is difficult to avoid in any nutritional epidemiologic study [30,31]. The meat intake frequency was only measured once at baseline; we could not evaluate the changes in dietary habits over the follow-up period. The unobserved changes in diet may lead to over- or underestimation of actual meat intake. Moreover, residual confounding still exists in our analyses.

## 5 Conclusions

This study’s findings suggest that a higher processed meat intake was associated with an increased risk of all-cause and cardiovascular mortality among frail individuals. Moderate intake of unprocessed red meat and higher intake of unprocessed poultry was associated with a decreased risk of all-cause, cancer, and cardiovascular mortality. Replacing processed meat or unprocessed red meat with fish or unprocessed poultry may be encouraged to reduce mortality and prolong life expectancy. These findings have important implications for dietary recommendations for people with frailty, which has been expanding rapidly due to population aging. Future studies are warranted to investigate further the health effects of different types of meat in frail individuals.

## Author contributions

JC contributed to conception and design. JC and WX contributed to writing the original draft and reviewing and editing the manuscript. JC and JT were involved in data curation and data analysis. EH contributed to reviewing and editing. JY and CW contributed to the conceptualization, methodology, project administration, and supervision and verified the underlying data. All authors gave final approval of the version to be published.

## Funding

The research results of this article are sponsored by the Kunshan Municipal Government Research Funding.

## Ethical statements

All participants signed an electronic consent, and the North West–Haydock Research Ethics Committee granted ethical approval to use the UK Biobank database (REC reference: 21/NW/0157).

## Conflict of interest

The authors have declared no conflicts of interest in this article.
