# Supplementary material for: Associations between meat consumption and all-cause and cause-specific mortality in middle-aged and older adults with frailty
Source: J Nutr Health Aging. 2024 Feb 14;28(4):100191. doi: 10.1016/j.jnha.2024.100191 (PMC12877277; doi:10.1016/j.jnha.2024.100191)
Supplement: Supplementary file 1 [file mmc1.docx]

**Supplement Materials**

eMethod. Assessment of meat consumption

Table S1. Operational definition of each of five criteria for creating the frailty phenotype score in the UK Biobank

Table S2. Components and categories of each meat group in the food frequency questionnaire (FFQ) and 24-hour dietary assessment

Table S3. Sensitivity analysis of the association between meat consumption and all-cause mortality among frail adults in the UK Biobank

**e Method** Assessment of meat consumption

We included the following meat-related items: oily fish, non-oily fish, unprocessed poultry, unprocessed pork, unprocessed beef, unprocessed lamb/mutton, and processed meat (e.g., bacon, assuage, and burger). Participants were asked how often they consumed each item (“never”, “less than once a week”, “once a week”, “2-4 times a week”, “5-6 times a week”, or “Once or more daily”). Following previous studies , consumption of oily and non-oily fish was added to create the fish category; consumption of unprocessed pork, beef, and lamb/mutton was summed to create the unprocessed red meat category. We assigned values for meat consumption according to the intake frequency per week: never eaten = 0, eaten <1 time/week = 0.5, 1 time/week = 1, 2-4 times/week = 3, 5-6 times/week = 5.5, and ≥1 time daily = 7. We further classified the intake frequencies for each meat type into four groups: 0-0.9 time/week, 1.0-1.9 times/week, 2.0-4.0 times/week, and >4.0 times/week.

We used the Oxford WebQ questionnaire , added to the assessment centres to assess more detailed dietary intake in the previous 24 hours to quantify the meat consumption. The Oxford WebQ questionnaire was administered online for a subgroup of UK Biobank participants every 3-4 months and repeated for four rounds over 16 months from February 2011 to June 2012. The Oxford WebQ participants were asked to select how many portions they consumed for each food item over the previous 24-hour period, with instructions specifying what one standard portion size represents (e.g., one serving of beef and one slice of ham). We calculated the daily intake in grams for each food item by multiplying the number of portions by the standard portion size. We then combined similar food items into distinct meat types to match the touchscreen questionnaire. A subsample of individuals (n=1,622) who completed at least three rounds of the Oxford WebQ data were included in the present study to estimate the mean intake of each meat type. These mean values were then assigned to the entire analytic sample.

**Table S1.** Operational definition of each of five criteria for creating the frailty phenotype score in the UK Biobank.

| **Criterion** | **Operational definition in the UK Biobank** |
| --- | --- |
| Weakness | Measured grip strength (sex and body-mass index adjusted cutoffs taken from Fried and colleagues) ^1^ |
| Slowness | Self-reported: “How would you describe your usual walking pace?” (response: slow=1, other=0) ^2^ |
| Exhaustion | Self-reported: “Over the past two weeks, how often have you felt tired or had little energy?” (response: more than half the days or nearly every day=1, other=0) ^2^ |
| Physical inactivity | Self-reported: UK Biobank physical activity questionnaire. We classified the responses into: none (no physical activity in the last 4 weeks), low (light DIY activity (e.g., pruning, watering the lawn) only in the past 4 weeks), medium (heavy DIY activity (e.g., weeding, lawn mowing, carpentry and digging), walking for pleasure, or other exercises in the past 4 weeks), and high (strenuous sports in the past 4 weeks) (response: none or light activity with a frequency of once per week or less=1, medium or heavy activity, or light activity more than once per week=0) ^3^ |
| Shrinking | Self-reported: “Compared with one year ago, has your weight changed?” (response: yes, lost weight=1, other=0) ^2^ |

Note: Frailty level was identified by the number of criteria met. Individuals with none were considered “robust/nonfrail”; those meeting one or two criteria were considered “prefrail”; and those with three to five criteria were defined as “frail”.

^1^ Definition used in the original description by Fried and colleagues.

^2^ Approximation based on available variables in UK Biobank assessment center data.

^3^ Definition used in the SHARE adaptation of the frailty phenotype.

**Table S2.** Components and categories of each meat group in the food frequency questionnaire (FFQ) and 24-hour dietary assessment.

| Meat type | Components in FFQ | Components in 24-h dietary assessment | Categories |
| --- | --- | --- | --- |
| Fish | Oily fish | oily fish | 0-0.9 time/week  1.0-1.9 times/week  2.0-4.0 times/week  >4.0 times/week |
|  | Non-oily fish | tinned tuna (non-oily fish) |  |
|  |  | breaded fish (non-oily fish) |  |
|  |  | battered fish (non-oily fish) |  |
|  |  | white fish (non-oily fish) |  |
| Unprocessed poultry | Poultry | poultry intake | 0-0.9 time/week  1.0-1.9 times/week  2.0-4.0 times/week  >4.0 times/week |
| Unprocessed red meat | Beef | beef | 0-0.9 time/week  1.0-1.9 times/week  2.0-4.0 times/week  >4.0 times/week |
|  | Lamb/mutton | lamb/mutton |  |
|  |  | pork |  |
| Processed meat | Processed meat | sausage | 0-0.9 time/week  1.0-1.9 times/week  2.0-4.0 times/week  >4.0 times/week |
|  |  | bacon |  |
|  |  | ham |  |
|  |  | crumbed or deep-fried poultry intake |  |
| Total meat | Processed meat  Unprocessed poultry  Unprocessed red meat | poultry intake, beef, lamb/mutton, pork, sausage  bacon, ham, crumbed or deep-fried poultry intake | 0-2.9 times/week  3.0-4.9 times/week  5.0-6.9 times/week  ≥7.0 times/week |

Abbreviation: FFQ, food frequency questionnaire.

**Table S3**. Sensitivity analysis of the association between meat consumption and all-cause mortality among frail adults in the UK Biobank.

|  | Exclude death occurred in the first year of follow-up (n=181) | | Exclude death occurred in the first two year of follow-up (n=376) | | Reprocessed missing values using multiple imputation method | | Additionally adjusted for Charlson Comorbidity Index | |
| --- | --- | --- | --- | --- | --- | --- | --- | --- |
|  | HR (95% CI) | P | HR (95% CI) | P | HR (95% CI) | P | HR (95% CI) | P |
| **Fish** |  |  |  |  |  |  |  |  |
| 0-0.9 time/week | Ref. |  | Ref. |  | Ref. |  | Ref. |  |
| 1.0-1.9 times/week | 0.99 (0.88, 1.11) | 0.797 | 0.94 (0.84, 1.05) | 0.289 | 0.93 (0.83, 1.04) | 0.205 | 0.98 (0.87, 1.09) | 0.694 |
| 2.0-4.0 times/week | 0.99 (0.88, 1.12) | 0.911 | 0.96 (0.85, 1.07) | 0.451 | 0.93 (0.83, 1.04) | 0.189 | 0.97 (0.87, 1.09) | 0.602 |
| > 4.0 times/week | 1.04 (0.88, 1.22) | 0.662 | 0.97 (0.83, 1.15) | 0.738 | 0.92 (0.79, 1.08) | 0.326 | 1.01 (0.86, 1.18) | 0.900 |
| per 25g/day | 1.01 (0.94, 1.09) | 0.773 | 0.99 (0.94, 1.05) | 0.860 | 0.97 (0.92, 1.03) | 0.33 | 0.99 (0.92, 1.07) | 0.837 |
| **Unprocessed poultry** | |  |  |  |  |  |  |  |
| 0-0.9 time/week | Ref. |  | Ref. |  | Ref. |  | Ref. |  |
| 1.0-1.9 times/week | 0.89 (0.81, 0.98) | 0.019 | 0.89 (0.80, 0.98) | 0.014 | 0.91 (0.84, 1.00) | 0.044 | 0.92 (0.84, 1.01) | 0.078 |
| 2.0-4.0 times/week | 0.83 (0.75, 0.91) | <0.001 | 0.82 (0.75, 0.91) | <0.001 | 0.84 (0.77, 0.91) | <0.001 | 0.85 (0.77, 0.93) | <0.001 |
| > 4.0 times/week | 0.65 (0.51, 0.82) | <0.001 | 0.64 (0.50, 0.82) | <0.001 | 0.69 (0.55, 0.87) | 0.001 | 0.71 (0.56, 0.89) | 0.004 |
| per 25g/day | 0.81 (0.74, 0.88) | <0.001 | 0.81 (0.74, 0.88) | <0.001 | 0.84 (0.78, 0.91) | <0.001 | 0.83 (0.76, 0.91) | <0.001 |
| **Unprocessed red meat** | |  |  |  |  |  |  |  |
| 0-0.9 time/week | Ref. |  | Ref. |  | Ref. |  | Ref. |  |
| 1.0-1.9 times/week | 0.86 (0.76, 0.98) | 0.019 | 0.83 (0.74, 0.94) | 0.004 | 0.85 (0.75, 0.96) | 0.008 | 0.86 (0.76, 0.97) | 0.015 |
| 2.0-4.0 times/week | 1.00 (0.88, 1.13) | 0.992 | 0.96 (0.85, 1.08) | 0.472 | 0.98 (0.87, 1.10) | 0.707 | 1.00 (0.88, 1.13) | 0.976 |
| > 4.0 times/week | 1.09 (0.94, 1.27) | 0.238 | 1.07 (0.93, 1.24) | 0.361 | 1.06 (0.92, 1.22) | 0.422 | 1.03 (0.89, 1.19) | 0.741 |
| per 25g/day | 1.07 (1.00, 1.14) | 0.046 | 1.07 (1.00, 1.14) | 0.049 | 1.07 (1.00, 1.13) | 0.041 | 1.05 (1.01, 1.10) | 0.014 |
| **Processed meat** | |  |  |  |  |  |  |  |
| 0-0.9 time/week | Ref. |  | Ref. |  | Ref. |  | Ref. |  |
| 1.0-1.9 times/week | 1.04 (0.95, 1.14) | 0.381 | 1.03 (0.94, 1.13) | 0.505 | 1.03 (0.94, 1.13) | 0.5 | 1.03 (0.95, 1.13) | 0.458 |
| 2.0-4.0 times/week | 1.13 (1.03, 1.23) | 0.009 | 1.12 (1.02, 1.23) | 0.013 | 1.10 (1.01, 1.19) | 0.034 | 1.10 (1.00, 1.20) | 0.039 |
| > 4.0 times/week | 1.22 (1.05, 1.40) | 0.007 | 1.24 (1.07, 1.43) | 0.004 | 1.21 (1.05, 1.38) | 0.007 | 1.18 (1.03, 1.36) | 0.019 |
| per 25g/day | 1.22 (1.09, 1.37) | 0.001 | 1.23 (1.09, 1.39) | 0.001 | 1.19 (1.06, 1.33) | 0.003 | 1.16 (1.03, 1.29) | 0.011 |
| **Total meat** | |  |  |  |  |  |  |  |
| 0-2.9 times/week | Ref. |  | Ref. |  | Ref. |  | Ref. |  |
| 3.0-4.9 times/week | 0.84 (0.62, 1.14) | 0.257 | 0.85 (0.63, 1.16) | 0.315 | 1.01 (0.64, 1.59) | 0.966 | 0.90 (0.67, 1.21) | 0.468 |
| 5.0-6.9 times/week | 0.86 (0.70, 1.06) | 0.167 | 0.83 (0.67, 1.03) | 0.085 | 0.91 (0.66, 1.26) | 0.575 | 0.90 (0.74, 1.11) | 0.338 |
| ≥7.0 times/week | 0.91 (0.74, 1.12) | 0.359 | 0.89 (0.72, 1.10) | 0.273 | 0.95 (0.69, 1.30) | 0.738 | 0.95 (0.78, 1.16) | 0.589 |
| per 25g/day | 1.00 (0.96, 1.04) | 0.905 | 1.00 (0.96, 1.04) | 0.945 | 1.00 (0.94, 1.06) | 0.944 | 1.00 (0.97, 1.04) | 0.838 |
